# Forgoing dental care for economic reasons in Switzerland: a six-year cross-sectional population-based study

**DOI:** 10.1186/1472-6831-14-121

**Published:** 2014-09-30

**Authors:** Idris Guessous, Jean-Marc Theler, Claire Durosier Izart, Silvia Stringhini, Patrick Bodenmann, Jean-Michel Gaspoz, Hans Wolff

**Affiliations:** Division of Primary Care Medicine, Department of Community Medicine, Primary Care and Emergency Medicine, University Hospitals of Geneva and Faculty of Medicine, University of Geneva, Geneva, Switzerland; Community Prevention Unit, University Institute of Social and Preventive Medicine, Lausanne University Hospital, Lausanne, Switzerland; Department of Epidemiology, Rollins School of Public Health, Emory University, Atlanta, USA; Vulnerable Population Unit, Department of Ambulatory Care and Community Medicine, University of Lausanne, Lausanne, Switzerland; Division of Penitentiary Medicine and Psychiatry, Department of Community Medicine, Primary Care, and Emergency Medicine, University Hospital Geneva and Faculty of Medicine, University of Geneva, Geneva, Switzerland; Unit of Population Epidemiology, Department of Community Medicine, Primary Care and Emergency Medicine, Geneva University Hospitals, 4 Rue Gabrielle-Perret-Gentil, 1211 Geneva 14, Switzerland

**Keywords:** Dental care, Forgoing, Socioeconomic status, Insurance

## Abstract

**Background:**

While oral health is part of general health and well-being, oral health disparities nevertheless persist. Potential mechanisms include socioeconomic factors that may influence access to dental care in the absence of universal dental care insurance coverage. We investigated the evolution, prevalence and determinants (including socioeconomic) of forgoing of dental care for economic reasons in a Swiss region, over the course of six years.

**Methods:**

Repeated population-based surveys (2007–2012) of a representative sample of the adult population of the Canton of Geneva, Switzerland. Forgone dental care, socioeconomic and insurance status, marital status, and presence of dependent children were assessed using standardized methods.

**Results:**

A total of 4313 subjects were included, 10.6% (457/4313) of whom reported having forgone dental care for economic reasons in the previous 12 months. The crude percentage varied from 2.4% in the wealthiest group (monthly income ≥13,000CHF, 1CHF ≈ 1$) to 23.5% among participants with the lowest income (<3,000CHF). Since 2007/8, forgoing dental care remained stable overall, but in subjects with a monthly income of <3,000CHF, the adjusted percentage increased from 16.3% in 2007/8 to 20.6% in 2012 (P trend = 0.002). Forgoing dental care for economic reasons was independently associated with lower income, younger age, female gender, current smoking, having dependent children, divorced status and not living with a partner, not having a supplementary health insurance, and receipt of a health insurance premium cost-subsidy.

**Conclusions:**

In a Swiss region without universal dental care insurance coverage, prevalence of forgoing dental care for economic reasons was high and highly dependent on income. Efforts should be made to prevent high-risk populations from forgoing dental care.

**Electronic supplementary material:**

The online version of this article (doi:10.1186/1472-6831-14-121) contains supplementary material, which is available to authorized users.

## Background

Oral health is a component of overall health, and oral illness is associated with several adverse health effects [1]. In meta-analyses, periodontis (i.e., chronic inflammation of structures that hold the tooth) has been associated with coronary heart disease [2], respiratory disease [3], and preterm birth [4]– although the evidence remains weak due to a limited number of low quality studies. There is also a growing body of literature linking oral illness to other chronic medical conditions such as type II diabetes, osteoporosis, Alzheimer’s disease and cognitive functioning [5–9].

The evidence for global oral health disparities (i.e., lower socioeconomic status being associated with greater oral disease) is well documented [10, 11], both in general adult and elderly populations [12–14]. The World Health Organization (WHO) has called for the reduction of inequity in oral health as one of its major worldwide goals [15].

With respect to the use of dental care, considerable socioeconomic inequalities at both the individual (intra-country) and collective (inter-country) levels across Europe and worldwide has been reported [16, 17]. Using data from the Swedish National Surveys of Public Health for 2004–2005, it has been shown that access to dental care explained 60% of the socioeconomic differential in oral health, while lifestyle factors explained only 29% of the gap [18].

Previous studies have identified several factors associated with the use and non-use of dental care [18–22]. Among these, the strongest independent factors of utilizing dental care appeared to be income and insurance [19, 20] with an effect that is probably modulated by the type of healthcare system. In several countries, healthcare systems do not cover (e.g., Switzerland) or only partially (e.g. France) cover dental care; any covered costs are generally limited to curative, but not preventive care [23, 24]. Thus, dental care costs are paid out-of-pocket on a fee-for-services basis to private dentists, a serious financial barrier to dental care, especially among economically vulnerable populations. In France, for example, the prevalence of the forgoing dental care for economic reasons has been shown to be higher in regions (i.e., departments) where dental procedure rates are highest [25]. This issue of access to dental care is of major concern because economically vulnerable populations are the most in need of this service [26, 27]. While the conceptual model for oral health inequalities is complex, differential access to dental care may contribute to oral health disparities, given that forgoing dental care due to financial hardship is associated with poorer oral health in several prior reports [28–32].

In Switzerland, dental insurance and direct dental care is not publicly subsidized, and thus it is probable that some individuals choose to forgo dental care. Until 2011, little was known about forgone healthcare for economic reasons in Switzerland. We have explored the importance of forgoing healthcare for economic reasons in Switzerland in two previous population-based studies [33, 34]; we found that one out of seven participants reported having forgone healthcare for economic reasons in the previous 12-month of the survey (2007–2010). This high prevalence was confirmed in a recent multicenter cross-sectional survey conducted among general practitioners working in the French-speaking part of Switzerland who enrolled a random sample of patients attending their private practices [35].

While specific population-based data on forgoing dental care for economic reasons are lacking in Switzerland, there is evidence that dental care is the most frequent type of forgone care [33, 34], and that meaningful socioeconomic disparities in oral health for both adults (e.g., periodontal disease) and children (e.g., dental caries) exist in Switzerland [36]. Accordingly, a recent popular initiative has been launched in two Swiss Cantons (Vaud: about 734,300 inhabitants; and Valais about 321,700 inhabitants) to implement a dental care insurance coverage that will be supported by part of the income tax (0.5% employee, 0.5% employer) [37].

In this aforementioned disparities context, and given the paucity of data on this topic in Switzerland, we aimed to determine the evolution, prevalence, and determinants of forgoing dental care for economic reasons in a Swiss population, over six years.

## Methods

### Surveyed population

We used data from the Bus Santé study [34]. The Bus Santé study is an ongoing cross-sectional population-based study that collects information on cardiovascular risk factors in the Canton of Geneva (Switzerland) [38]. The Canton of Geneva is a French-speaking urban state that differs from other cantons of Switzerland by several features, such as population density (including medical density), GDP per capita, education, proportion of foreign subjects, and unemployment (Additional file 1: Table S1). Subjects are selected independently throughout each year to represent the canton’s non-institutionalized adult residents. Eligible subjects are identified using a list of residents established by the local government. This listing includes all potential eligible participants except persons living illegally in the country. Exclusion criteria for the Bus Santé are 1) age <35 or >75 year old or 2) being institutionalized. Stratified random sampling is used to select participants by gender within each five-year age stratum, selecting a number of participants that is proportional to the corresponding population distribution. Each participant receives several self-administered, standardized questionnaires. There are no language restrictions, as long as the participants are able to understand and answer the questionnaires. The 2007–2012 participation rates varied between 58% and 62%. Participants from the six waves of the annual surveys were combined and used as the surveyed population, except in the estimation of the annual trends.

The Bus Santé study was approved by the ethical research committee of the Geneva University Hospitals (10-030R) and all study participants provided written informed consent.

### Variables

#### Forgoing dental care for economic reasons

Two variables were used to measure economic barriers to dental care. Participants were asked, “*During the previous 12 months, have you forgone any healthcare for economic reasons*?” “*If yes, which type of care?:*” If any were checked off, participants specified which one(s) among the list of 12 non-mutually exclusive types of care forgone; one of these was “dental care.” Respondents who answered “yes” and “dental care” were considered as having forgone dental care for economic reasons within the previous year. These questions are different yet very similar to questions used in prior published reports [20, 28, 39, 40]. Of note, information on the type of dental care (e.g., preventive) forgone was not collected.

### Demographics and socioeconomic factors

Participants were grouped by age: <45 years, 45–64 years, and ≥65 years. Citizenship was categorized as Swiss or non-Swiss. Information on marital status and dependent children at home (age <15 years) was collected. Based on previous research [34], we considered having children aged <15 years as a better predictor of forgoing care than “having children in general” because children aged <15 years do not have a regular income. Occupational position was categorized into “high” (self-employed and/or non-manual [i.e., person who performs professional, managerial, or administrative work such as teacher, journalist, salesperson, nurse) and “*low*” (salaried and/or manual [i.e., person whose occupation requires manual labor such as forestry worker, factory worker, plumber]), education level (high [≥13 years] and low [<13 years]), and monthly household income (<CHF 3000/month, 3000–4999, 5000–6999, 7000–9499, 9500–12999, ≥13000) were used as indicators of socioeconomic status. If the individual was retired, information on his/her last occupation was used to define his/her occupational position category.

### *Health*insurance status and smoking status

In Switzerland, citizens can supplement their compulsory basic *health* insurance with a supplementary *health* insurance. Supplementary *health* insurance rarely includes dental care in Switzerland. Yet, we hypothesized that the presence or absence of supplementary insurance was associated with the use of dental care: subscribing to a supplementary insurance (even one that does not cover dental care) may highlight a personal health behavior towards preventive healthcare access - including oral health - that is independent of income. This is in line with previous works from Manski et al. [41, 42]. In addition, it is possible that supplementary insurance discounts the price of certain health services and thus improves the ability to pay for other services like dental care [43, 44]. Participants were therefore asked whether they had supplementary *health* insurance (yes *vs.* no) and whether they received a *health* insurance premium cost subsidy from the state (yes *vs.* no). *S*pecific information about dental care insurance was not available. We considered smoking status as it has been previously associated with oral health and access to dental care [28, 45]. Information on smoking status was self-reported and defined as current smoker versus not smoker.

### Statistical analyses

The means and frequencies (%) of study variables were calculated. Analyses were performed using Student’s t-tests or ANOVA for continuous data, and chi-square tests for categorical data. Measures of deviation were reported as standard deviation (SD) or 95% confidence intervals (95% CI). The Cochran-Armitage trend test was used to test trends across survey periods. The Cochran-Armitage test checks for a trend in binomial proportions across levels of a single factor. Logistic regression models were used to test associations between forgoing dental care and study variables, adjusting for survey year when appropriate. To test for temporal trends in forgoing dental care while accounting for potential changes in the study population structure (e.g. education), adjusted prevalence of forgoing dental care were estimated for each survey year and non-parametric trend tests were performed. Overall and monthly household income-specific prevalence were adjusted for age, sex, smoking status, occupational position, marital status, dependent children at home (age <15 years), education, Swiss citizenship, complementary health insurance, receiving health insurance premium subsidy, and monthly household income (only for overall prevalence analysis). Temporal trends were tested for overall prevalence and by monthly household income. Variables included in the models were selected *a priori* given their reported or potential influence on oral health and/or forgoing health care. Trends were tested for overall prevalence and by monthly household income. The collection of forgoing dental care information in the Bus Santé study was introduced in mid-2007. Thus, due to the small number of participants with information on forgoing dental care collected in 2007, survey years 2007 and 2008 were combined. Each attribute (including smoking) was considered both as a determinant of forgoing dental care for economic reasons and as a potential confounder of the associations between other attributes included in the models and forgoing dental care for economic reasons. Only individuals for whom all covariates of interest for the purpose of this study were available were included in the analysis. All p-values were 2-tailed with significance set at <0.05 (<0.10 for interaction test). All analyses were performed using SAS software (SAS Institute, Inc., Cary, North Carolina) and Stata 12 (College Station, TX: StataCorp LP).

## Results

Among the 4,584 participants of the Bus Santé study, a total of 4,313 subjects (50% women) were included in the analysis (Table 1). The main reason for participants to be excluded from the analysis was missing data on income (N = 259, 5.7%). The numbers of participants included in the analysis for each survey year were as follows: 749 (17.4%) in 2007/8, 1006 (23.3%) in 2009, 944 (21.9%) in 2010, 883 (20.5%) in 2011, and 740 (17.2%) in 2012. The overall mean age was 51.9 yr (SD, 10.9). Except for education level, the prevalence of the different characteristics considered did not differ significantly across survey years.

**Table 1:** **Participants’ characteristics (Bus Santé, N = 4313, Geneva, Switzerland), overall and by survey year**

	Period or survey year						
Characteristics	2007–2012	2007/8	2009	2010	2011	2012	P value
Number of participants	4313	740	1006	944	883	740	
Mean age (SD)	51.9 (10.9)	51.6 (11.0)	51.5 (10.8)	52.3 (11.1)	51.5 (10.8)	52.7 (11.1)	0.11
Age category N (%)							0.11
Age <45y	1371 (31.8)	246 (33.2)	330 (32.8)	293 (31.0)	290 (32.8)	212 (28.6)	
Age 45–64y	2219 (51.4)	384 (51.9)	525 (52.2)	476 (50.4)	451 (51.1)	383 (51.8)	
Age ≥65y	723 (16.8)	110 (14.9)	151 (15.0)	175 (18.5)	142 (16.1)	145 (19.6)	
Female gender N (%)	2155 (50.0)	358 (48.4)	517 (51.4)	470 (49.8)	448 (50.7)	362 (48.9)	0.72
High education level N (%)	1941 (45.0)	327 (44.2)	418 (41.6)	411 (43.5)	413 (46.8)	372 (50.3)	**0.004**
Household monthly income N (%)							0.33
<3,000CHF	276 (6.40)	42 (5.7)	58 (5.8)	72 (7.6)	54 (6.1)	50 (6.8)	
3,000–4,999CHF	644 (14.9)	103 (13.9)	158 (15.7)	142 (15.0)	126 (14.2)	115 (15.5)	
5,000–6,999CHF	792 (18.4)	146 (19.7)	172 (17.1)	172 (18.2)	171 (19.4)	131 (17.7)	
7,000–9,499CHF	920 (21.3)	162 (21.9)	228 (22.7)	214 (22.7)	174 (19.7)	142 (19.2)	
9,500–12,999CHF	834 (19.3)	143 (19.3)	193 (19.2)	176 (18.6)	158 (17.9)	164 (22.2)	
≥13,000CHF	847 (19.6)	144 (19.5)	197 (19.6)	168 (17.8)	200 (22.6)	138 (18.6)	
Swiss citizenship N (%)	3002 (69.6)	541 (73.1)	693 (68.9)	653 (69.2)	612 (69.3)	503 (68.0)	0.23
Current smokers N (%)	906 (21.0)	160 (21.6)	227 (22.6)	192 (20.3)	178 (20.2)	149 (20.1)	0.62
Independent/non-manual N (%)	2377 (55.1)	408 (55.1)	526 (52.3)	521 (55.2)	505 (57.2)	417 (56.3)	0.26
Having dependent children (<15y) at home N (%)	1431 (33.2)	223 (30.1)	356 (35.4)	307 (32.5)	297 (33.6)	248 (33.5)	0.23
Marital status N (%)							0.36
Single	450 (10.4)	83 (11.2)	99 (9.0)	106 (11.2)	78 (8.8)	84 (11.3)	
Married or in a relationship	2815 (65.3)	486 (65.7)	675 (67.1)	617 (65.4)	569 (64.4)	468 (63.2)	
Divorced, not in a relationship	671 (15.6)	120 (16.2)	154 (15.3)	139 (14.7)	146 (16.5)	112 (15.1)	
Divorced, in a relationship	112 (2.6)	16 (2.2)	26 (2.6)	23 (2.4)	26 (2.9)	21 (2.8)	
Widow/er, not in a relationship	242 (5.6)	28 (3.8)	50 (5.0)	54 (5.7)	60 (6.8)	50 (6.8)	
Widow/er, in a relationship	23 (0.5)	7 (0.9)	2 (0.2)	5 (0.8)	4 (0.45)	5 (0.7)	
Supplementary health insurance status N (%)	1938 (44.9)	312 (42.2)	442 (43.9)	423 (44.8)	415 (47.0)	346 (46.8)	0.26
Premium cost subsidized N (%)	632 (14.6)	106 (14.3)	135 (13.4)	133 (14.1)	145 (16.4)	113 (15.3)	0.41

Statistically significant P values (<0.05) are highlighted in bold.

Table 2 reports univariate and multivariate association of participant’s characteristics with forgoing dental care for economic reasons. In univariate analysis, forgoing dental care for economic reasons was associated with age, gender, education, household monthly income, smoking status, Swiss citizenship, and occupational position. Divorced participants who were not living with a partner had the highest prevalence of forgoing dental care but most confidence intervals overlapped across marital status categories. Participants with dependent children at home had forgone dental care more frequently than participants without dependent children. Participants without supplementary health insurance were more likely to forgo dental care than participants with such supplementary insurance. The prevalence of forgoing dental care was higher in the presence of premium subsidies than in their absence.

**Table 2:** **Prevalence (%, 95% CI) and multivariate associations (Odds Ratio, 95% CI) of participants’ characteristics with forgoing dental care for economic reasons in the 2007–2012 Geneva (N = 4313) Bus Santé study (Switzerland)**

	Univariate analyses		Multivariate analyses*
	% (95% CI) forgoing dental care for economic reasons	P value	OR (95% CI)
**Characteristics**			
Age category		**<0.001**	
Age <45y	12.4 (10.7–14.3)		**2.52 (1.64–3.85)**
Age 45–64y	11.2 (9.9–12.6)		**2.48 (1.70–3.64)**
Age ≥65y	5.3 (83.7–7.1)		1.00 (Ref)
Gender		**0.005**	
Female	11.9 (10.6–13.4)		**1.26 (1.03–1.56)**
Male	9.3 (8.1–10.6)		1.00(Ref)
Education level		**0.018**	
High	8.4 (7.2–9.7)		0.97 (0.77–1.22)
Low	12.4 (11.1–13.8)		1.00 (Ref)
Household monthly income		**<0.001**	
<3,000CHF	23.5 (18.7–29.0)		**9.78 (5.23–18.28)**
3,000–4,999CHF	17.0 (14.2–20.2)		**5.00 (2.80–8.91)**
5,000–6,999CHF	13.2 (11.0–15.8)		**4.30 (2.45–7.54)**
7,000–9,499CHF	11.0 (9.0–13.2)		**3.70 (2.15–6.37)**
9,500–12,999CHF	6.7 (5.1–8.6)		**2.24 (1.26–3.99)**
>13,000CHF	2.4 (1.4–3.6)		1.0 (Ref)
Smoking status		**<0.001**	
Current smoking	17.7 (15.2–20.3)		**1.71 (1.37–2.13)**
No current smoking	8.7 (7.8–9.7)		1.00 (Ref)
Citizenship		**<0.001**	
Swiss citizenship	9.4 (8.5–10.6)		0.87 (0.70–1.09)
No Swiss citizenship	13.1 (11.3–15.1)		1.00 (Ref)
Occupational position		**<0.001**	
High	9.6 (8.4–10.8)		1.12 (0.88–1.43)
Low	11.8 (10.4–13.3)		1.00 (Ref)
Dependent children (<15y) at home		**0.006**	
Having dependent children (<15y) at home	12.4 (10.8–14.3)		**1.31 (1.03–1.66)**
Not having dependent children (<15y) at home	9.7 (8.6–10.8)		1.00 (Ref)
Marital status		**<0.001**	
Divorced, not in a relationship	17.6 (14.8–20.7)		**1.29 (1.01–1.67)**
Other			1.00 (Ref)
Single	11.8 (8.9–15.1)		
Married or in a relationship	8.8 (7.8–9.9)		
Divorced, in a relationship	9.8 (5.0–16.9)		
Widow/er, not in a relationship	10.7 (7.1–15.3)		
Widow/er, in a relationship	0.1 (0.0–14.8)		
Supplementary health insurance status		**<0.001**	
Complementary health insurance	4.8 (3.9–5.9)		**0.45 (0.35–0.58)**
No complementary health insurance	15.3 (13.8–16.8)		1.00 (Ref)
Premium cost subsidy status		**<0.001**	
Premium subsidized	21.5 (18.4–24.9)		**1.47 (1.14–1.90)**
No premium subsidized	8.7 (7.8–9.8)		1.00 (Ref)

*Adjusted for survey year in addition to all variables listed in the table.

Statistically significant P values (<0.05) are highlighted in bold.

**Figure 1:**
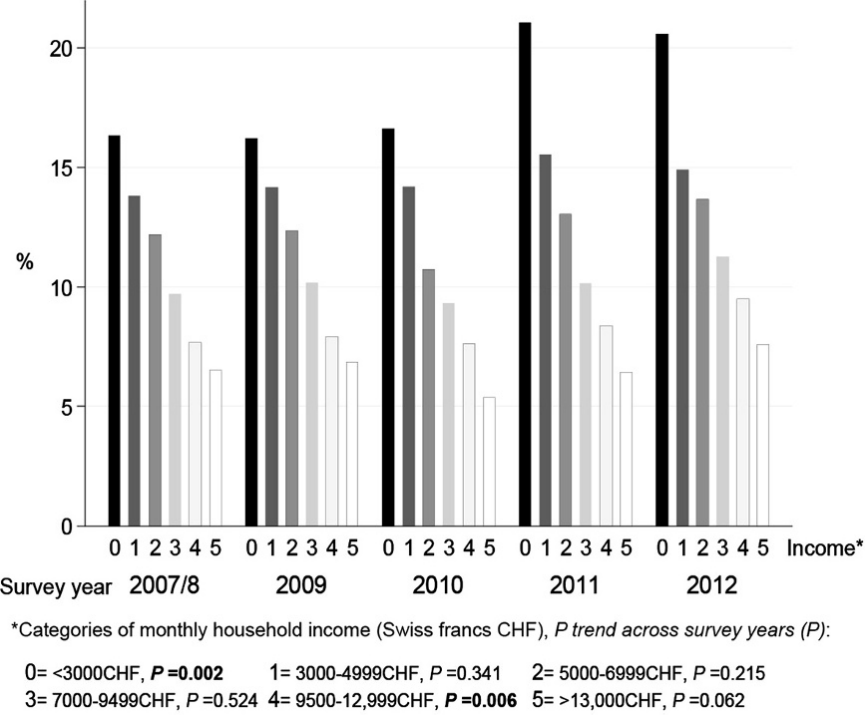
**Adjusted prevalence of forgoing dental care for economic reasons (%), by survey year and monthly household income (Swiss francs CHF).** Footnote: prevalence adjusted for age, sex, smoking status, occupational position, marital status, dependent children at home (age <15 years), education, Swiss citizenship, supplementary health insurance, and health insurance premium cost subsidy. Statistically significant P values for trend (<0.05) are highlighted in bold.

The adjusted odds of forgoing dental care for economic reasons by specific characteristics are also presented in Table 2. Women had higher odds of forgoing dental care than men. The odds of forgoing dental care increased with decreasing monthly household incomes (P for trends < 0.05); participants in the lowest income category had almost 10 times the odds of participants in the highest income category of forgoing dental care (OR = 9.8 95% CI 5.2–18.3). Participants with dependent children at home, and divorced participants living without a partner had higher odds of forgoing dental care than other participants. Having a supplementary health insurance and receiving a premium cost subsidy, respectively, decreased and increased the odds of forgoing dental care.The adjusted overall prevalence of forgoing dental care did not differ significantly between 2007/8 and 2012 (10.6% in 2007/8 and 11.6% in 2012, p trend value = 0.089). Among participants with a monthly household income < 3000CHF, the adjusted prevalence increased from 16.3% in 2007/8 to 20.6% in 2012 (P for trends = 0.002), while it did not differ significantly among participants with greater monthly household income (Figure 1).

## Discussion

Using population-based data from a Swiss urban region with a compulsory *health* insurance system but without a universal dental care insurance coverage, we found that forgoing dental care for economic reasons was frequently reported. This is the first study providing evidence of inequalities in financial barriers to dental care in the general adult population in Switzerland. The mean proportion of forgoing dental care for economic reasons was, in general, lower than estimates reported in studies conducted in other countries [46, 47]. But, the proportion of forgoing dental care for economic reasons specifically among participants with the lowest income was very close to estimates reported in Canada and France [20, 28, 40], for example. Using data from the 2007/09 Canadian Health Measures Survey, Thompson et al. found that 17.3 percent of respondents had avoided a dental professional because of cost within the previous year [20]. We observed an increasing trend among respondents with the lowest income, a trend also observed in the Australian general adult population between 1994 and 2008 [46]. The high proportion of dental care forgone, and the rising trend among the poorest is worrisome– not only because oral health is associated with a better quality of life, but also in light of the growing evidence linking oral health to chronic disease like cardiovascular disease, respiratory disease and osteoporosis [1, 2, 6, 7, 48]. Of note, the role that dental health access and visits play within the relationship between oral health and chronic disease is yet to be determined.

### Income and other factors associated with forgoing dental care

Socioeconomic status is inversely associated with forgoing dental care [10, 11]. In our study, monthly household income had a linear negative association with the forgoing of dental care for economic reasons, confirming results from previous studies [18, 20–22]. In Canada for example, where dental care is not publicly covered for the general population, Thompson et al. recently showed that compared to respondents with the highest income, respondents with lower incomes were over four times more likely to avoid a dental professional because of cost [20]. Similar results were previously found by others using different data sources in Canada [21, 22]. Our findings highlight that persons with lower income in Switzerland – who are at greater risk of oral illness [26, 27] – also forgo dental care more frequently. This stresses the major role of income in the utilization of dental care in privately financed dental care systems [20]. Given the current paucity of data, further studies should be conducted in Switzerland to examine the implications of dental treatment costs for low-income families and/or the working poor, similarly to studies conducted in other settings [47, 49].

Several factors positively associated with forgoing healthcare for economic reasons in this study are in line with previous reports [20, 28, 40–42]. In particular, after taking into account other factors, not having a supplementary *health* insurance doubled the odds of forgoing dental care for economic reasons. This is very similar to findings reported in France [28, 40], and is in line with a phenomena called “unobserved behavior” by Manki et al. This study reported that having medical insurance with or without coverage for dental care in a non-universal care healthcare system (e.g., United States) is associated with an increased likelihood of having a dental visit [41, 42]. Including information about type of medical coverage in these regression models in future studies could improve the accuracy of any associations [41].

In contrast to previous studies [28, 40], but coinciding with a recent survey [20], we found no independent association between education level and forgoing dental care for economic reasons, after adjustment for income. While our reasons for the absence of independent association between education and forgoing care are speculative, it has been suggested that more educated individuals may be more sensitive to the need to use money for other priorities, and therefore decline more treatment as a result [20]. A similar mechanism has been suggested to explain the fact that women are more likely to forgo dental care for economic reasons when compared to men (a result observed in our study, as well as in a recent previous Canadian study [20] and other previous European reports [28, 40]). The lack of association with occupational position can be explained, at least in part, by the fact that dental insurance is not provided by employers in Switzerland.

### Trends in forgoing dental care

Overall, the prevalence of forgoing dental care for economic reasons remained statistically stable between 2007 and 2012, but increased among participants with the lowest income. Our data suggests that the prevalence of forgoing dental care disproportionately increased during these six years among participants with household monthly income <3,000CHF. The global financial crisis of 2008 and resulting economic downturn in 2009 stalled demand for exports and put Switzerland in a recession [50] in 2009. The Canton of Geneva ranks first in Switzerland with respect to the level of unemployment, which increased from 6.0% in 2007/8 to 6.8% in 2009 and to 7.0% in 2010 [51]. A previous report suggests that the importance of cost as a risk factor for forgoing healthcare increases during an economic downturn [52]. Our data shows that the 2008–9 economic downturn may have been harmful for those with the fewest economic resources.

### Dental insurance

The current initiative in two Swiss cantons proposes to improve access to dental care, particularly for low income people, by implementing a publicly funded dental care insurance coverage [37]. We lack information about dental care insurance. Dental insurance is a major determinant of dental utilization [19, 20, 22]. The impact of insurance seems to be independent of income, as suggested by the fact that regardless of income level, the insured utilize more dental care than the uninsured [20].

In the context of discussing an implementation of dental care insurance, previous observations suggest that oral health inequalities nevertheless persist in systems with dental care insurance coverage [10, 53, 54]. This can be attributable, at least in part, to limitations within the dental care insurance coverage policies [20]. Low reimbursement rates and lack of a full year coverage for some insurance programs (e.g. Medicaid) are reasons why implementing a dental insurance may not have the expect impact on visits and/or oral health [20]. Income-related inequality persists even in the presence of dental insurance coverage, as observed by Duncan et al using data from the 2009 Canadian Health Measures Survey [55]. The lack of availability of practitioners can of course also mitigate the impact of a dental insurance. Thus, if the current initiative conducted in the two Swiss cantons passes, the quality of the dental insurance that would be proposed in the bill should be carefully examined.

Several factors not directly related to insurance such as smoking, low perception of medical need, language and communication problems, and other psychosocial factors also contribute to oral health inequalities in and access to dental care [27, 56]. Recent findings from the life-course approach also suggest that a considerable proportion of inequalities around regular dental attendance are already established in childhood and persists throughout the life-course [57]. Inequalities in adults may then be relatively unresponsive to contemporaneous health policy interventions such as the implementation of dental insurance [57]. In addition, difficulties in managing vulnerable patients with high dental needs and lack of compliance with regular care have been expressed by private dentists [27]. Finally, concerns have been informally raised about the overwhelming demand that could follow after the introduction of a universal dental insurance, the administrative burden, as well as the economic viability and sustainability of such dental care insurance coverage. Thus, in the perspective of introducing a publicly funded dental care insurance that might improve access to dental care in general and among the most vulnerable patients in particular, one should also consider sustainable solutions that incorporate the complicated myriad of contributing factors, rather than just address financial need. One is reminded that dental care access is not the sole reason for oral health inequalities (e.g. smoking and oral health inequalities). Overall, dental care insurance coverage is probably a *necessary but not sufficient* action to eliminate oral health inequalities.

### Dental insurance and global dental health strategy

Forgoing dental care is associated with poorer oral health [28–32]. A very recent study published in this *Journal* showed that Canadians from the general adult population who reported cost barriers to dental care had more untreated decay, missing teeth, poorer oral health and more frequent oral pain [30]. Those reporting cost barriers also had a higher prevalence of needing dental treatment [30]. Currently, the Swiss dental care system is privately financed and a meaningful proportion of the adult general population is potentially at risk of forgoing dental care. While income is a key determinant of utilizing dental care, implementing a universal dental insurance is thought to be the most feasible approach to improve, at the population level, the access to dental care. Such universal dental care insurance should be characterized by conditions (e.g, low cost-sharing, income-based sliding scale for premiums) that assure equity in access to dental care [30, 55, 58]. Two cantons in Switzerland have recently launched an initiative to implement a dental care insurance coverage that will be supported by income taxes [37]. We suggest that in order to better design this universal insurance, relevant information on dental need and use of dental care should be first collected among a representative sample in the two cantons. For example, reasons for dental non-attendance are various and considerable differences among populations exist as suggested by a recent study conducted among Europeans over the age of 50 [56]. In Switzerland, the most frequently named reason for non attending dental visit was “not considered to be necessary” [56]. This suggests that efforts to provide more information about the benefits of regular dental attendance are sorely needed. Integrated into a global oral health strategy [59], dental insurance impact could be tested at a population level in an experimental manner.

### Strengths and limitations

It should be noted that the study participants are representative of the general population contrary to studies conducted in dental offices or hospitals, and that the large sample size allows conclusions to be drawn for an entire Swiss canton. However, our results may not be representative of Switzerland as a whole, even if it is very likely that results in other Swiss urban cantons such as Basel or Zurich would be similar. Our analyses are also limited by the self-reported nature of the information collected. The survey included dental care without further information on the nature and/ or severity of dental care (e.g., preventive *versus* curative/rehabilitative services). We did not consider psychosocial, communication, or cultural determinants of forgoing dental care for economic reasons as these characteristics were not collected in the surveys. Participants were asked whether they had supplementary *health* insurance and whether they received a premium cost subsidy from the state; whether these supplementary health insurances or premium cost subsidies covered, at least in part, dental care costs was unknown. Also, the absence of follow-up surveys precluded us from disentangling the amount of dental care forgone and delayed. Because we aimed to determine the independent associations between dependent children and marital status with forgoing dental care for economic reasons, we did not divide income by consumption units (such as the number of persons depended on the reported household income).

## Conclusions

Oral health is part of an individual’s general health, and organization of the public health system should ensure equity in access to health care, including dental care. Our findings suggest that, in Switzerland, dental care is not equally accessible across society. Socioeconomically disadvantaged individuals who are known to be at higher risk of oral disease frequently forgo dental care for economic reasons. Efforts should be made to prevent high-risk populations from forgoing dental care.

## Electronic supplementary material

Additional file 1: Table S1: Main (2012) comparisons of the surveyed population (State of Geneva) and the entire Swiss population. **Table S2.** Adjusted prevalence of forgoing dental care for economic reasons (%), by survey year and monthly household income (Swiss francs CHF). (DOCX 17 KB)

## Abbreviations

SD: Standard deviation
WHO: World Health Organization.
