# Supplementary material for: Forgoing dental care for economic reasons in Switzerland: a six-year cross-sectional population-based study
Source: BMC Oral Health. 2014 Sep 30;14:121. doi: 10.1186/1472-6831-14-121 (PMC4190381; doi:10.1186/1472-6831-14-121)
Supplement: Supplementary file 1 — Additional file 1: Table S1: Main (2012) comparisons of the surveyed population (State of Geneva) and the entire Swiss population. Table S2. Adjusted prevalence of forgoing dental care for economic reasons (%), by survey year and monthly household income (Swiss francs CHF). (DOCX 17 KB) [file 12903_2014_451_MOESM1_ESM.docx]

**Additional file 1**

**Table S1.** Main (2012) comparisons of the surveyed population (State of

Geneva) and the entire Swiss population

|  | **State of Geneva** | **Switzerland** |
| --- | --- | --- |
| Inhabitants | 463 101 | 8 039 060 |
| Population density per km² | 1 884,0 | 201,0 |
| Non Swiss % | 39,7 | 23,3 |
| Age categories % |  |  |
| 0-19 years | 21,4 | 20,4 |
| 20-64 years | 62,3 | 62,2 |
| 65 years or more | 16,4 | 17,4 |
| Urban population % | 99,2 | 73,7 |
| Speaking language % |  |  |
| German | 5,0 | 64,9 |
| French | 80,8 | 22,6 |
| Italian | 7,1 | 8,3 |
| English | 10,7 | 4,6 |
| Surface km² | 282,4 | 41 285,0 |
| Unemployment % | 5,46 | 3,16 |
| GDP per capita in Swiss francs | 104 914 | 73 947 |
| Social subsidies % | 5,3 | 3,1 |
| Private physicians per 100 000 inhabitants | 361 | 210 |
| Hospital beds for 1000 inhabitants | 5,7 | 4,8 |
| High education % | 39,3 | 29,8 |
|  |  |  |
| Statistics from the Swiss Office of Statistics [**http://www.bfs.admin.ch/bfs/portal/fr/index/regionen/kantone/ge/key.html**](http://www.bfs.admin.ch/bfs/portal/fr/index/regionen/kantone/ge/key.html) | | |

**Table S2.** Adjusted prevalence of forgoing dental care for economic reasons (%), by survey year and monthly household income (Swiss francs CHF)

|  | Survey year | | | | | |  |
| --- | --- | --- | --- | --- | --- | --- | --- |
|  | All survey years | 2007/8 | 2009 | 2010 | 2011 | 2012 | P value for year trend |
| All household monthly income | 10.9 (10.6-11.1) | 10.6 (10.0-11.3) | 10.6 (1.0-11.1) | 10.8 (10.2-11.4) | 10.7 (10.0-11.3) | 11.6 (11.0-12.2) | 0.089 |
| <3,000CHF | 18.3 (17.0-19.6) | 16.3 (13.3-19.3) | 16.2 (13.6-18.8) | 16.6 (14.1-19.2) | 21.1 (17.6-24.5) | 20.6  (17.9-23.3) | **0.002** |
| 3,000-4,999CHF | 14.5 (13.8-15.3) | 13.8 (11.9-15.7) | 14.2 (12.7-15.6) | 14.2 (12.4-15.9) | 15.5 (13.7-17.4) | 14.9 (13.1-16.7) | 0.341 |
| 5,000-6,999CHF | 12.4 (11.8-12.9) | 12.2 (10.9-13.5) | 12.4 (11.3-13.4) | 10.7 (9.5-11.9) | 13.0 (11.7-14.4) | 13.7 (12.3-15.0) | 0.215 |
| 7,000-9,499CHF | 10.1 (9.7-10.5) | 9.7 (8.7-10.7) | 10.2 (9.3-11.0) | 9.3 (8.4-10.2) | 10.1 (9.1-11.2) | 11.3 (10.0-12.5) | 0.524 |
| 9,500-12,999CHF | 8.3 (7.9-8.6) | 7.7 (6.9-8.5) | 7.9 (7.2-8.6) | 7.6 (6.9-8.4) | 8.4 (7.6-9.1) | 9.5 (8.6-10.4) | **0.006** |
| >13,000CHF | 6.5 (6.2-6.8) | 6.5 (5.7-7.3) | 6.8 (6.2-7.5) | 5.4 (4.8-5.9) | 6.4 (5.8-7.0) | 7.6 (6.8-8.4) | 0.062 |

Footnote: prevalence and P values for year trend are adjusted for age, sex, smoking status, job position, marital status, dependent children at home (age <15 years), education, Swiss citizenship, complementary health insurance, and health insurance premium subsidy. Statistically significant P values for trend (<0.05) are highlighted in bold.
